# Hypoxia-Inducible Factor and Oxidative Stress in Tendon Degeneration: A Molecular Perspective

**DOI:** 10.3390/antiox13010086

**Published:** 2024-01-10

**Authors:** Hamzah Shahid, Vivek Kumar Morya, Ji-Ung Oh, Jae-Hyung Kim, Kyu-Cheol Noh

**Affiliations:** 1 Dongtan Sacred Heart Hospital, Hallym University, Hwaseong-si 18450, Gyeonggi-do, Republic of Koreajaehkim11@gmail.com (J.-H.K.); 2 School of Medicine, Hallym University, Chuncheon City 24252, Gangwon-do, Republic of Korea

**Keywords:** tendon disorders, hypoxia, oxidative stress, tendinopathy, degenerative disorders

## Abstract

Tendinopathy is a debilitating condition marked by degenerative changes in the tendons. Its complex pathophysiology involves intrinsic, extrinsic, and physiological factors. While its intrinsic and extrinsic factors have been extensively studied, the role of physiological factors, such as hypoxia and oxidative stress, remains largely unexplored. This review article delves into the contribution of hypoxia-associated genes and oxidative-stress-related factors to tendon degeneration, offering insights into potential therapeutic strategies. The unique aspect of this study lies in its pathway-based evidence, which sheds light on how these factors can be targeted to enhance overall tendon health.

## 1. Introduction

Tendons are specialized connective tissues anchored between muscles and bones that serve as mechanical bridges to transmit forces from muscles to bones and vice versa [1]. The extracellular matrix of tendons contains a higher amount of collagen than that of muscles, providing intensive tensile strength and facilitating locomotion. The arrangement of collagen and other supportive proteins allows tendons to resist large loads and mechanical strain. At strain levels between 4% and 8%, tendons progressively become easier to extend, although their length still returns to its original value. Strain values between 6% and 8% lie within the normal physiological range; however, even within this range, microscopic degeneration within the tendon may occur, especially with repeated and/or prolonged stressing. Beyond 8% to 10% strain, macroscopic failure occurs owing to intrafibril damage as a result of molecular slippage [2,3]. The tendon matrix is composed of 70–80% collagen 1, with other components, including collagen III, collagen V, collagen XII, elastin, aggrecan, proteoglycans, and glycolipids, playing important roles in maintaining the homeostasis of the tendon [4].

Tendons can withstand high mechanical loads; however, excessive loading can result in tendon overuse injuries. Chronic overuse injuries are the result of damage accumulation over multiple load cycles and not just a single overload event [5,6]. Chronic overuse can result in microtrauma, and, without adequate healing, it may lead to tendinopathy or even rupture. The underlying mechanisms of tendinopathy are not well understood, but it is believed to be caused by a combination of factors, such as increased levels of matrix metalloproteinases (MMPs), the downregulation of tissue inhibitors of matrix metalloproteinases (TIMPs), and the upregulation of collagen 3 relative to collagen 1, leading to an aberrant extracellular matrix (ECM) [7,8,9]. This can further alter the mechanical environment of the tendon and perpetuate its degeneration.

Tendinopathy is a significant concern, as it is estimated to account for 30% of musculoskeletal disorders [10] and is highly prevalent in older individuals [11]. To better understand the etiology of tendinopathy, it is divided into three stages: reactive tendinopathy, failed healing, and degeneration [12]. Tendinopathy is accompanied by pain, swelling, functional limitations, and anatomical changes within the tendon, which can ultimately lead to tendon rupture if left untreated [13].

Tendinopathy is a multifactorial disease with both intrinsic and extrinsic risk factors. The intrinsic risk factors include age, sex, genetics, weight, and health conditions, while the extrinsic risk factors include repetitive loading, medications, hypoxia, and oxidative stress [14]. However, the role of physiological factors such as hypoxia and oxidative stress in tendon disorders requires further elucidation [15].

Tendons are sparsely vascularized tissues that are adapted to low oxygen concentrations. Following initial tendon injury, a low oxygen concentration promotes the overexpression of hypoxia-inducible factor (HIF-1), which can regulate various cellular processes, including apoptosis, inflammation, and neo-vascularization, suggesting that hypoxia may play an important role in tendon degeneration [7,16,17,18].

Oxidative stress, caused by an imbalance between ROS and antioxidants, is another physiological condition implicated in various musculoskeletal disorders [15]. Tendon injury leads to a state of oxidative stress, marked by an increase in ROS species, which upregulate pro-inflammatory cytokines that promote apoptosis and extracellular matrix degradation [19]. Antioxidants such as Vitamin C, Curcumin, Nicotinamide Mononucleotide (NMN), and Superoxide Dismutase (SOD) [20,21,22,23] have been reported to promote tendon repair, but their exact mode of action requires further exploration [20]. This review discusses the role of hypoxia and oxidative stress in the pathogenesis of tendinopathy and provides a molecular perspective on novel targets that may be responsible for poor tendon healing.

## 2. Role of HIF-1 in Tendons

HIF-1 is a crucial regulatory protein that controls oxygen homeostasis and plays a primary role in managing the balance between oxygen supply and demand [24]. Under low-oxygen conditions, the hypoxic environment triggers the synthesis of HIF-1, which then regulates angiogenesis, erythropoiesis, and glycolysis to restore oxygen equilibrium [25]. To comprehend the oxygen requirements necessary to govern the physical environment of a particular tissue, it is imperative to understand oxygen tension in the tendon [26,27]. Tendons are highly avascular tissues with oxygen concentrations estimated to be between 1 and 5% [26,28,29]. This limited vasculature can result in hypoxia accompanying tissue damage, making tendinopathy treatment more challenging [16]. Research has established that multiple factors contribute to chronic tendon disorders, and among them, HIF-1 may play a role in mediating tendinopathy pathogenesis [16,30]. Previous studies have shown that HIF-1 is significantly upregulated in tendinopathic tendons, where it regulates the expression of pro-inflammatory cytokines, apoptotic mediators, and angiogenesis, exacerbating tendinopathy [16,30,31].

A well-documented impairment of the HIF-1/vascular endothelial growth factor (VEGF) pathway has been observed in chronic tendon disorders. In tendinopathy, due to elevated hypoxia, HIF-1α expression is upregulated, leading to the promotion of neo-angiogenesis [32]. Angiogenesis, mediated by VEGF, plays a crucial role in tendon healing, particularly during acute tendon injuries [30,32]. However, in chronic tendon injuries, persistently high VEGF expression can result in the formation of hypertrophic scars and keloids, the stimulation of MMPs, and the inhibition of TIMPs [30,32,33,34]. Furthermore, hypervascularization may lead to excessive degradation of the ECM, which can be detrimental to tendon health [33]. Prolonged VEGF upregulation in a hypoxic environment may lead to excessive scarring, which results in altered ECM homeostasis and excessive ECM degradation, ultimately leading to collagen accumulation [16,33,35]. Based on these factors, we speculated that hypoxia-induced and VEGF-mediated neo-angiogenesis may play a significant role in tendinopathy pathogenesis [16,30,31,33,35]. Currently, there is limited research on the role of impaired hypoxia-related genes in chronic tendon disorders. Nonetheless, the available in vitro, in vivo, and clinical studies support the idea that impaired hypoxia persists throughout tendinopathy. Therefore, the regulation of impaired hypoxia may be a valuable strategy for promoting tendon healing.

### 2.1. HIF-1α Is Degraded under Normoxia

**Figure 1:**
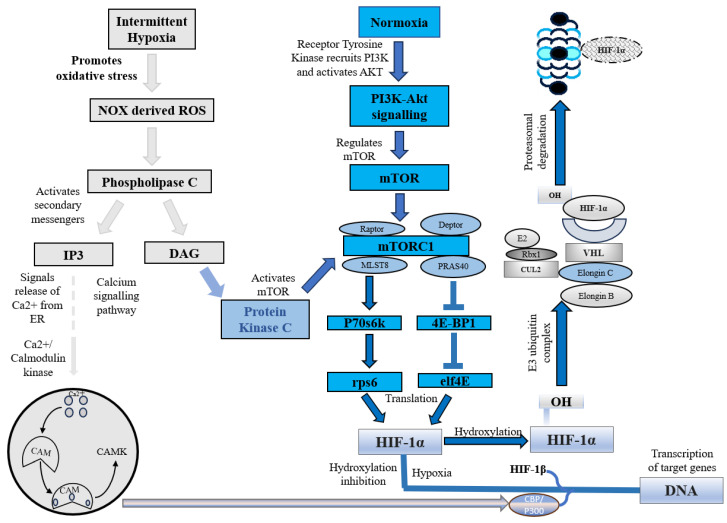
HIF-1α under intermittent hypoxia and normoxia. NOX, NADPH oxidase; ROS, Reactive oxygen species; IP3, Inositol 1,4,5-trisphosphate; DAG, Diacylglycerol; CAM, Calcium/calmodulin; CAMK, Ca^2+^/calmodulin-dependent protein kinase; mTOR, Mammalian target of rapamycin; PI3K, Phosphoinositide 3 kinase; AKT, Protein kinase B; mTORC1, Mammalian target of rapamycin complex 1; MLST8, Mammalian lethal with SEC13 protein 8; PRAS40, Proline-rich Akt substrate of 40 kDa; P70s6k, Ribosomal protein S6 kinase beta-1; eIF4E, Eukaryotic translation initiation factor 4E; 4E-BP1, Eukaryotic translation initiation factor 4E binding protein 1; rps6, Ribosomal protein S6; HIF-1α, Hypoxia-inducible factor 1-alpha; OH, Hydroxide; CUL2, Cullin-2; Rbx1, Ring-Box 1; E2, Ubiquitin-conjugating enzymes; E3, E3 ubiquitin ligases; VHL, Von Hippel–Lindau.

Under normoxic conditions, HIF-1α is constantly synthesized and degraded via ubiquitin-mediated proteolysis [36]. However, under hypoxic conditions, HIF-1α rapidly accumulates owing to a decrease in its degradation rate. HIF-1α is hydroxylated by prolyl hydroxylases (PHD1, PHD2, PHD3, and PHD4), which are necessary for its degradation. PHD2 primarily hydroxylates HIF-1α/2α, whereas PHD3 is associated with HIF-2α hydroxylation [36,37]. Following prolyl hydroxylation, HIF-1α was recognized by the E3 ubiquitin ligase complex formed by the von Hippel–Lindau tumor suppressor protein (pVHL), Elongin B/C, Cullin-2, and RING-box protein 1 (Rbx1). This recognition triggered ubiquitination of HIF-1α by the ligase complex, leading to its proteasomal degradation. (Figure 1) [37].

In addition to this canonical pathway, a non-canonical pathway for HIF-1α inhibition is mediated by a factor that inhibits hypoxia-inducible factor (FIH). Under normoxic conditions, FIH inhibits HIF-1α by hydroxylating an asparagine residue (Asn803 in human HIF-1α and Asn851 in human HIF-2α) within the C-terminal transactivation domains of HIF-1α and 2α. This reduces the activity of the CTAD domain and cooperates with p300/CBP, a histone acetyltransferase [38]. However, under hypoxic conditions, the FIH activity is inhibited, allowing HIF-1 to regulate the transactivation of target genes [39].

### 2.2. HIF-1 Expression under Hypoxic Conditions

**Figure 2:**
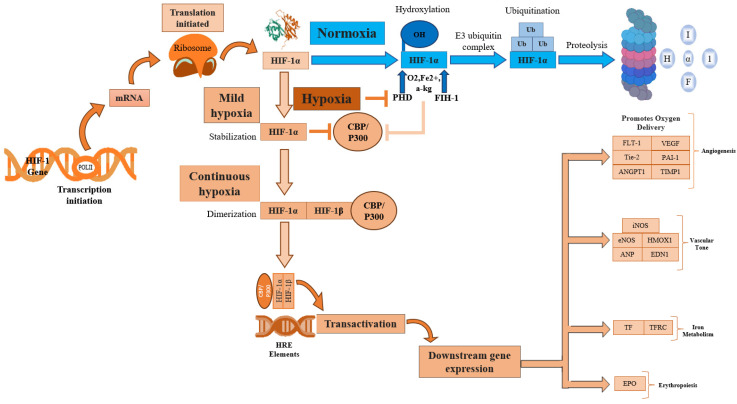
HIF-1α activation regulates several key cellular processes. HIF-1α, Hypoxia-inducible factor 1-alpha; HIF-1β, Hypoxia-inducible factor 1-beta; POLIII, Polymerase 3; CBP/P300, Histone acetyltransferases CBP and p300; p300, E1A binding protein; CBP, CREB-binding protein; mRNA, Messenger RNA; PHD, Prolyl hydroxylase domain; FIH-1, Factor inhibiting hypoxia-1; HRE, Hypoxia response element; Ub, Ubiquitination; OH, Hydroxide; O_2_, Oxygen; Fe^2+^, Iron(II) ion; α-KG, α-ketoglutarate; EPO, Erythropoietin; TF, Transferrin; TFRC, Transferrin receptor; iNOS, Inducible nitric oxide synthase; eNOS, Endothelial nitric oxide synthase; HMOX1, Heme Oxygenase; ANP, Atrial natriuretic peptide; END1, Endothelin1; FLT1, Fms related receptor tyrosine kinase 1; VEGF, Vascular endothelial growth factor; ANGPT1, Angiopoietin-1; Tie-2, Angiopoietin-1 Receptor; PAI-1, Plasminogen activator inhibitor 1; TIMP-1, Tissue inhibitor of metalloproteinases.

Hypoxia, a condition characterized by a lack of adequate oxygen supply, is known to play a critical role in regulating both tissue and cell fate. The primary regulator of hypoxia is hypoxia-inducible factor, specifically the constitutively expressed subunit HIF-1β and the oxygen-regulated subunit HIF-1α. In response to hypoxia, HIF-1α is hydroxylated and accumulates in the cytosol before being transported to the nucleus, where it dimerizes with HIF-1β [40]. This dimer then binds to hypoxia response elements (HREs) within oxygen-regulated genes [41], enabling the activation of genes involved in angiogenesis, erythropoiesis, and the regulation of cell survival, proliferation, and metabolism (Figure 2) [25,41,42]. Therefore, its expression is crucial for the regulation of several key molecular pathways.

### 2.3. Upregulation of HIF-1 in Animal Tendinopathy Models

Animal models of tendinopathy have been proven to be effective in replicating the disease condition (Table 1). These models show a cellular milieu similar to that observed in tendinopathy. Zhang and colleagues reported that elevated levels of alarmins (produced by injured tissues) were present in a rotator cuff impingement mouse model. Additionally, HIF-1α, an important alarmin, was found to be significantly increased at both the gene and protein levels [43]. Sahin et al. developed a rat patellar tendinosis model to investigate the effects of HIF-1α-mediated VEGF angiogenesis on the biomechanical properties of tendons. Researchers have observed that HIF-1α overexpression during tendinopathy promotes VEGF expression. The overexpression of HIF/VEGF may lead to the breakdown of the surrounding tissue through VEGF-regulated MMP release, resulting in a decrease in the tendon’s mechanical strength. Researchers have proposed that regulating neo-angiogenesis may be a potential treatment for tendinopathy [30].

One potential strategy to promote tendon repair may be to modulate the levels of HIF-1α following injury (Table 1) (Figure 2) [34]. HIF-1α has been found to be upregulated in tendinopathy [16,30,31], and previous studies have suggested that HIF-1α inhibition can alleviate tendinopathy symptoms (Table 1) [34,44,45]. In a recent study conducted by Jiao et al., YC-1, an inhibitor of HIF-1, was evaluated for its potential in promoting tendon repair. These results demonstrate that YC-1 treatment effectively promoted tendon healing by inhibiting HIF-1 and inflammation. Moreover, YC-1 treatment was found to increase the expression of tendon markers Tenascin C (TNC) and Scleraxis (SCX) [44]. Similar findings were observed by Wang et al., who found that the HIF-1α levels were elevated in a rat tendinopathy model induced by prostaglandin E2. Their study suggested that the dysregulation of HIF-1/hedgehog signaling may play a role in tendinopathy. Treatment with an HIF inhibitor, Asperosaponin VI (ASA VI), promoted tendon repair via the upregulation of tenogenesis-associated markers such as SCX, TNC, collagen 1, and Mohawk X(MKX), and the downregulation of tendinopathy markers MMP, VEGF-A, and KDR. ASA VI treatment also reduced the expression of Hgg factors, including Shh, Ptch1, Gli1, and Sox 9. Researchers have proposed that in an anaerobic environment, HIF-1α may induce hedgehog signaling, but ASA VI may promote tendon repair by inhibiting HIF-1α and Shh expression in an autocrine manner [45]. The positive effects of HIF-1 inhibition in attenuating other musculoskeletal diseases (such as rheumatoid arthritis and osteoarthritis) have been reported [46,47]. Meng and colleagues evaluated the efficacy of AMSP-30m (a HIF-1α inhibitor) in alleviating arthritic symptoms in an adjuvant-induced arthritis rat model. The results showed that AMSP-30m treatment induced anti-arthritic effects through its anti-angiogenic and anti-inflammatory activity, suggesting that HIF-1α regulation may serve as a treatment for rheumatoid arthritis [46]. Additionally, Casticin was found to alleviate inflammation in a knee osteoarthritis rat model. Casticin may improve knee osteoarthritis by inhibiting hypoxia and due to its anti-inflammatory capacity [47]. These findings indicate that the proper regulation of HIF-1α may be a viable strategy for the treatment of various musculoskeletal diseases, including tendinopathy (Table 1).

### 2.4. Effect of HIF-1 under Differential Hypoxia on Tenocyte/TDSC Proliferation In Vitro

To simulate hypoxic conditions in vitro, researchers have employed various methods, such as the use of chemicals (ascorbic acid or Cobalt Chloride) and hypoxia induction incubators (Anaeropack) (Table 1). These models have the potential to uncover the effects of differential hypoxia on tendon cell populations [26,29]. Liang et al. evaluated the effects of total hypoxia (0.1%) on human tenocytes. They observed a significant increase in the levels of HIF-1α and hypoxia-induced apoptosis after 48 h. This increase in apoptosis was attributed to the upregulation of the hypoxia-related pro-apoptotic proteins BNIP3 (BCL2/adenovirus E1B 19 kDa protein-interacting protein 3) and NIX (BCL2/adenovirus E1B 19 kDa protein-interacting protein 3-like). Researchers have suggested that poor healing of the tendon may be due to increased cellular apoptosis [31]. While hypoxia is well documented as inhibiting cell proliferation, it also serves a physiological function for stem cells, including tendon-derived stem cells (TDSCs). TDSCs, the resident stem cell population at the tendon, are considered a viable therapeutic option for the treatment of chronic tendon disorders due to their tenogenic lineage differentiation capacity [48].

An in vitro study demonstrated that a low oxygen concentration of 2% promotes the proliferation and lineage differentiation of TDSCs, as opposed to normoxic conditions of 20%. The study also found that TDSCs exhibited higher clonogenicity and proliferative capacity under low oxygen concentrations [49]. These findings were supported by another study conducted by Zhang et al., who tested the multi-differentiative potential of TDSCs under different hypoxic conditions. The study found that a low oxygen concentration of 5% was optimal for promoting the self-renewal and differentiation of TDSCs into adipocytes, chondrocytes, and osteocytes. Additionally, researchers observed that hypoxia promotes the expression of stem cell and tenocytic markers [28,29]. Another study conducted by Yu et al. also reported that a low oxygen concentration of 5% was optimal for promoting the self-renewal capacity of human TDSCs [50]. The enhanced proliferative and differentiative capacity under low-oxygen conditions can provide a means to rapidly expand the TDSC population and evaluate the effects of hypoxia in an in vitro model [29,50].

### 2.5. HIF-1 Impairment Promotes Tendinopathy

Following tendon injury, there is an increase in the levels of hypoxia-associated genes; therefore, an increase in HIF-1α expression is now considered a biomarker of early tendinopathy [16]. HIF-1α overexpression has been shown to induce structural alterations, upregulate apoptotic genes, and promote the expression of inflammatory cytokines, leading to mitochondrial dysfunction, increased calcification, and tissue degeneration [30,31,51].

Józsa and colleagues observed hypoxia-induced structural alterations in the tendinopathic tissues of tendinopathy patients [51]. A recent study reported that hypoxia can induce mitochondrial structural alteration, leading to mitochondrial dysfunction [52]. Furthermore, hypoxia can promote osteochondrogenic differentiation and tissue calcification, a well-documented feature of tendinopathy [53].

**Table 1:** Studies depicting the effects of downregulation and upregulation of hypoxia in various models. ↑: upregulation; ↓: downregulation.

	Study Purpose	Reported Outcome	Expression	Significant Findings	Ref.
Upregulated	Role of HIF-1 in early tendinopathy. Impact of hypoxia on cellular apoptosis, inflammatory cytokine expression, and matrix regulation in tenocytes in vitro.	HIF-1 plays a critical role in early tendinopathy. In vitro, it may promote cellular apoptosis and inflammation and alter matrix collagen composition.	HIF-1α ↑ IL-8 ↑ IL-6 ↑ Bcl-2 ↑ Caspase3 ↑ Caspase 7 ↑ HSP70 ↑	Hypoxia has a significant impact on the regulation of inflammatory and apoptotic signals within tendon cells, leading to changes in the collagen matrix synthesis. It has been suggested that hypoxia may also regulate oxidative damage through the MAPK pathway. Furthermore, elevated levels of hypoxia have been shown to negatively impact the collagen composition by increasing the synthesis of collagen 3.	[16]
	Determine the expression of HIF-1α and hypoxia-associated apoptotic gene Bnip3 during different stages of tendinopathy	Hypoxia precedes Bnip3-induced apoptosis. Apoptosis increases with worsened rotator cuff health.	HIF1α ↑ Bnip3 ↑	It is postulated that an excessive amount of programmed cell death, triggered by hypoxia, may result in reduced cellular proliferation, a decline in cell numbers, and the transformation of cells into chondroid-like cells as the rotator cuff deteriorates further. The regulation of apoptotic protein production may play a significant role in promoting the healing response during the repair process.	[17]
	The study aims to understand the role of tendinopathy-associated hypoxia and its influence on VEGF production, MMP expression, and the biomechanical properties of the tendon in the course of tendinopathy in a rat patellar tendon healing model	Tendon biomechanical properties are impaired during tendon healing.	HIF1α ↑ VEGF ↑ MMP3 ↑	Tendinopathy has been found to result in the upregulation of HIF-1α, which in turn activates the production of VEGF, leading to angiogenesis. This process, involving HIF-1/VEGF, contributes to the repair of the tendon but weakens its mechanical strength by promoting the breakdown of the surrounding tissue through VEGF-induced MMPs. MMPs are responsible for degrading the tendon ECM. The study has established a correlation over time between HIF-1/VEGF-induced angiogenesis, MMP-3-related tissue restructuring, and reduced biomechanical properties during tendon healing. These findings suggest that regulating VEGF levels to modulate neo-angiogenesis may provide a potential treatment approach for tendinopathy.	[30]
	Determine the effects of self-induced extreme hypoxia on tenocytes	Increased expression of HF-1a regulates apoptosis via Bnip3 and VEGF following hypoxia exposure.	HIF1α ↑ Bnip3 ↑ VEGF ↑	The elevated expression of HIF-1α was found to be positively correlated with increased levels of hypoxia-induced apoptotic genes, such as Bnip3 and VEGF. VEGF is crucial for the normal repair and remodeling processes, but an adequate vascular supply is essential for the successful outcome of these processes.	[31]
	Determine the expression of alarmins in murine tendinopathy model	Alarmins such as HIF-1α are significantly upregulated.	HIF1α ↑ S100A9 ↑	Tendon biopsies obtained from tendinopathy patients reflect a chronic disease state (symptomatic patient), which is not reflective of an early disease state. Alarmins may regulate the early stages of tendinopathy. Tendinopathy is accompanied by elevated HIF-1α, HIF-1α may promote tenocyte apoptosis in vitro.	[43]
	Identifying potential serological biomarkers for monitoring the effectiveness of treatment in rotator cuff disease	VEGF, HIF-1α, and MMP were elevated in rotator cuff disease.	HIF1α ↑ VEGF ↑ MMP ↑	It has been demonstrated through a systematic review that the utilization of distinct biochemical entities (such as HIF-1α, VEGF, ANGPT, MMP) may hold significant clinical applications in monitoring the status of rotator cuff disease and aiding in the implementation of effective management strategies.	[54]
	Evaluate the levels of alarmins pre- and post-treatment in diseased versus healthy tendons	Levels of alarmins such as HIF-1α and S100A9 were elevated in diseased tendons. However, the levels were significantly reduced in the post-treatment group.	Pre-treatment HIF-1α ↑ S100A9 ↑ HMGB ↓ IL33 ↓ Post-treatment HIF-1α ↓ S100A9 ↓ HMGB ↑ IL33 ↑	HIF-1α has been implicated in the regulation of alarmins such as S100A9, HMGB1, and IL-33 in inflammatory disorders. As a result, it is possible that HIF-1α may also play a similar role in the tendon tissue. Further investigation is necessary to determine the specific contributions of S100A9, HMGB1, and IL-33 to inflammation in tendon diseases, as well as their potential to aid in tendon healing.	[55]
Downregulated	Evaluate the effect of HIF-1α inhibition using YC-1 in an in-vivo and in-vitro tendinopathy model	HIF-1α inhibitor (YC-1) promoted tendon repair by downregulation of inflammatory cytokines and MMPs and promoting the expression of tenocyte markers.	YC-1 Treatment HIF1α ↓ IL-6 ↓ MMP-3 ↓ MMP-9 ↓ MMP-13 ↓ TNC ↑ SCX ↑	The suppression of HIF-1α resulted in the amelioration of tendinopathy and the promotion of tendon repair. HIF-1 is implicated in the pathogenesis of TP through its involvement in the NF-κB and MAPK signaling pathways. This is supported by the observation that the administration of YC-1 led to a decrease in inflammatory factors, such as IL-6, and ECM mediators, such as MMP-13. Furthermore, the upregulation of tenocyte markers, including TNC and SCX, following YC-1 treatment suggests improved tendon healing.	[44]
	Effect of HIF-1 inhibitor (Asperosaponin VI) in a rat tendinopathy model	Asperosaponin promoted tendon repair by downregulation of HIF-1α and hedgehog signaling pathway. Indicated by increased expression of tendonogenesis-associated markers such as Scx, Mkx, EYA1, EYA2 mRNA, collagen 1, and TNC.	ASA VI treatment HIF1α ↓ MMP ↓ VEGF ↓ KDR ↓ SCX ↑ TSC ↑ COLLAGEN 1 ↑ MKX ↑	The application of ASA VI resulted in a decrease in mRNA levels for MMP3, VEGF-A, KDR, and VWF in Achilles tendon tissues, thereby ameliorating the pathological vascular environment. This decrease in mRNA levels was associated with a reduction in HIF-1α, which in turn decreased the expression of hedgehog signaling genes in an autocrine manner. As a result, the expression of Ptch1 was reduced, Smo inhibition was enhanced, and the expression of Gli proteins in the nucleus was reduced. These effects collectively inhibited the hedgehog pathway and decreased the expression of the osteogenic marker SOX9. This decrease in SOX9 expression may prevent TDSCs from differentiating into the osteogenic lineage and promote repair.	[45]

VEGF, Vascular endothelial growth factor; MMP, Matrix metalloproteinases; HIF-1α, Hypoxia-inducible factor 1-alpha; S100A9, S100 calcium-binding protein A9; ECM, Extracellular matrix; BNIP3, BCL2/adenovirus E1B 19 kDa protein-interacting protein 3; HMGB1, High mobility group box 1 protein; IL-33, Interleukin-33; ANGPT, Angiopoietin; IL-8, Interleukin-8; IL-6, Interleukin-6; Bcl-2, B-cell lymphoma/leukemia-2; Hsp70, Heat shock protein 70; TNC, Tenascin C; SCX, Scleraxis; YC-1, Lificiguat; MAPK, Mitogen-activated protein kinase; NF-κB, Nuclear factor kappa B; MMP-3, Matrix Metalloproteinase 3; MMP-9, Matrix Metalloproteinase 9; MMP-13, Matrix Metalloproteinase 13; KDR, Kinase insert domain receptor; MKX, Mohawk X; EYA1, Eyes absent homolog 1; EYA2, Eyes absent homolog 2; SOX9, SRY-Box Transcription Factor 9; ASA VI, Asperosaponin VI.

As previously mentioned, the overexpression of HIF-1α is well documented in human tendinopathy [16,43]. Benson and colleagues compared the expression of HIF-1 and BNIP3 in rotator cuff tendinopathy patients against controls and found a marked increase in the levels of HIF-1α and BNIP3 in all patients compared to the controls. This leads to excess cellular apoptosis due to BNIP3 regulation, resulting in poor healing of the tendon [17]. In another study, the levels of HIF-1 and VEGF were found to be elevated in samples of rotator cuff tendon disease, and there was also a marked increase in the levels of MMPs, which promotes matrix degradation [54]. Mosca et al. compared the levels of HIF-1α pre- and post-tendinopathy treatment and found that the levels of HIF-1α were increased in diseased tendons when compared to post-treatment and healthy tendons (Table 1) [55]. Millar and colleagues also reported an increase in HIF-1α levels in tendinopathy samples, which may promote the expression of pro-inflammatory cytokines and various MMPs, disturbing ECM homeostasis and leading to degeneration [16]. These studies suggest that an impaired HIF-1-mediated pathway may contribute to tendon degeneration, and targeting the components of this pathway may be a viable treatment for tendon-related disorders.

### 2.6. Regulating the Hypoxic Environment May Promote Tendon Repair

Because a hypoxic environment is a physiological condition for various stem cell niches, including embryonic and adult somatic stem cells, researchers have utilized hypoxic preconditioning to promote the proliferative and differentiative capacity of various stem cells, including mesenchymal stem cells (MSCs), adipose-derived mesenchymal stem cells (AD-MSCs), bone-marrow-derived stem cells (BMSCs), and TDSCs [29,49,50,56]. These studies have documented that hypoxic preconditioning also promotes the expression of tenogenic markers in these stem cells [57,58,59]. The transplantation of these stem cells, particularly those pre-conditioned under hypoxic conditions, has been reported to be effective in treating chronic tendon disorders. For example, a study found that hypoxia-preconditioned BMSCs showed improved histological scores and biomechanical properties in a rabbit tendon injury model compared with normoxic BMSCs [57]. Another study expanded upon the previous findings to determine how transplanted cells migrated and functioned following engrafting. Hypoxic MSCs labeled with super magnetic iron oxide were found to migrate into the punch area of the supraspinatus tendon and engraft into the injured tendon. The labeled cells were detectable even four weeks post-transplantation and significantly improved biomechanical strength and enhanced recovery in a rat rotator cuff tear model [60]. Thus, hypoxic pre-conditioning plays an important role in mediating stem cell differentiation, and the transplantation of hypoxic stem cells significantly improves the histological score and biomechanical properties [49,58,59,60]. However, the exact mechanism by which hypoxia mediates differentiation potential remains unknown [57], and further research is needed to determine whether it functions as an inducer of other growth factors.

**Figure 3:**
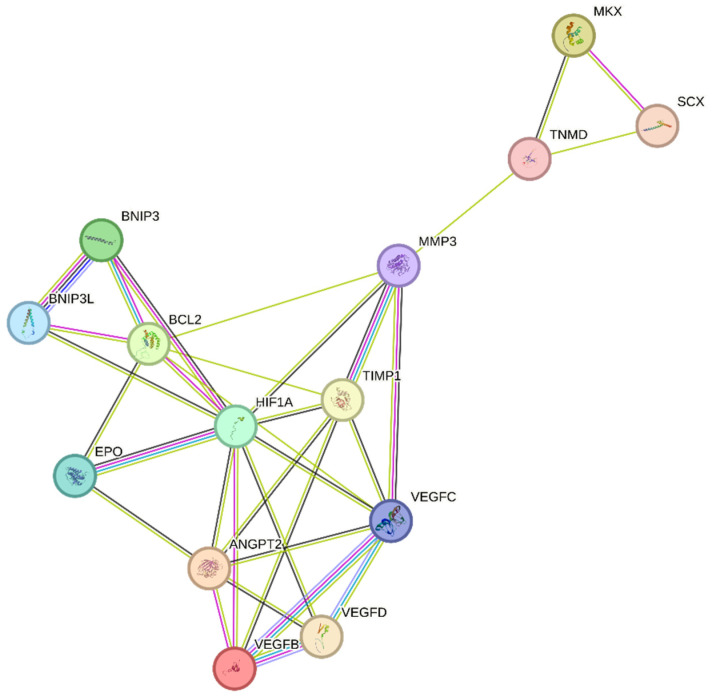
Regulation of the hypoxia factor HIF-1α may promote tendon repair in chronic tendon disorders. HIF-1α, Hypoxia-inducible factor 1-alpha; BCL2, B-cell lymphoma/leukemia-2; SCX, Scleraxis; MMP-3, Matrix Metalloproteinase 3; MKX, Mohawk X; EPO, Erythropoietin; ANGPT2, Angiopoietin 2; VEGFD, Vascular endothelial growth factor D; VEGFC, Vascular endothelial growth factor C; VEGFB, Vascular endothelial growth factor B; BNIP3, BCL2/adenovirus E1B 19 kDa protein-interacting protein 3; BNIP3L, BCL2/adenovirus E1B 19 kDa protein-interacting protein 3-like; TIMP1, Tissue inhibitor of metalloproteinases 1; TNMD, Tenomodulin.

Unfortunately, tendinopathy is a complex, multifactorial disorder with an uncertain etiology [61]. However, the increased expression of the hypoxia marker HIF-1 following tendon injury may play a critical role in its initiation [16]. We speculate that regulating the expression of HIF-1 and its target genes, as shown in Figure 3, may help to attenuate tendinopathy severity. One possible mechanism by which HIF-1 may contribute to tendinopathy is the regulation of MMP3 by its activator TIMP-1 [62]. Exposure to hypoxia increases HIF-1α levels, which in turn activates several key pathways, including MMPs and their inhibitors, TIMPs. TIMP-1, in particular, plays an important role in regulating MMP levels, as a balance of MMP/TIMP is necessary for maintaining tendon homeostasis. An imbalanced MMP/TIMP ratio caused by dysregulated TIMP-mediated MMP regulation may lead to impaired remodeling during tendon repair [63,64]. TIMP-1 is known to play a role in various biological processes, including tissue remodeling, promoting growth factor activity, and wound healing [64]. As an MMP inhibitor, TIMP-1 primarily targets and inhibits MMP-9. However, it also has a strong affinity for MMP-3. The MMP3–TIMP1 complex is formed by interactions between the catalytic domains of human stromelysin-1 (MMP-3) and human TIMP-1 [65,66]. Further research is necessary to fully understand the role of HIF-1 and TIMP-1 in tendinopathy and to develop effective treatments for this debilitating condition [63,66].

Previous research has indicated that TIMP-1 hinders excessive matrix degradation by impeding MMP activity, especially that of MMP-9, as MMP-2 and MMP-9 are upregulated in tendinopathies [63]. Consequently, the inhibition of MMP-2 and MMP-9 by their respective TIMPs can promote tendon repair. In addition to MMP-2 and MMP-9, MMP-3 has been found to be dysregulated during tendinopathies, which is inconsistent with its expected role in matrix remodeling. A study also suggested that the expression of MMP-3 is suppressed during tendinopathy, and MMP-3 polymorphism may contribute to chronic tendon disorders [67]. As MMP-3 also plays a crucial role in matrix remodeling, the downregulation or complete inhibition of MMP-3 by TIMP-1 would imply the failure of proper matrix remodeling [68]. We speculate that following tendon rupture, elevated HIF-1α increases TIMP-1 expression, which in turn inhibits MMP-3. This inhibition of MMP-3 may lead to the altered expression of tenocytic markers, such as SCX, MKX, and TNMD. This suggests that MMP-3 may be involved in the maintenance of tenocytic homeostasis. Therefore, a possible treatment strategy may be to balance the regulation of the TIMP-1/MMP-3 complex. Further studies focusing on the regulation of this complex following tendon injury may be a viable treatment strategy to promote tendon repair and may lead to increased expression of tenocytic proliferative markers (SCX, MKX, and TNMD) (Figure 3).

Recently, the hypoxia-associated apoptotic gene BNIP3 has been linked to tendinopathy [17,31]. A study of patients with subacromial impingement and rotator cuff tears revealed a significant increase in HIF-1α levels. This overexpression leads to the buildup of fragmented DNA in tissue samples, suggesting that HIF-1α promotes apoptosis in hypoxic environments. Researchers have proposed that the upregulation of the apoptotic gene BNIP3 leads to apoptosis of fibroblasts and fibroblast-like cells, resulting in reduced collagen synthesis and a hypoxic environment [17]. Therefore, maintaining a dynamic balance between pro-apoptotic and anti-apoptotic factors in a hypoxic environment is crucial for effective tissue healing [17,69]. This is further supported by another study, which found that cells lacking HIF-1 showed a reduced expression of BNIP3 and underwent lower apoptosis, indicating that BNIP3 is a direct target of HIF-1α [70]. The apoptosis associated with tendinopathy results in reduced cell proliferation and increased chondrogenic metaplasia, a hallmark of tendinopathy [17,31]. The manipulation of BNIP3 expression following HIF-1α upregulation may be a potential treatment for tendinopathy (Figure 3).

In recent years, VEGF has garnered considerable attention as a potential therapeutic target for promoting tendon repair. Following tendon injury, hypoxia induces VEGF expression, thereby promoting increased vascularization (Table 1) [30,32]. However, in tendinopathy, VEGF expression remains elevated during the remodeling phase, impeding the tendon repair process. This prolonged VEGF-induced angiogenesis leads to the upregulation of matrix metalloproteinases (MMPs) and the suppression of tissue inhibitors of metalloproteinases (TIMPs), resulting in extracellular matrix (ECM) degradation and collagen 1 downregulation, which negatively impact tendon repair [30,71]. Moreover, in a fully healed tendon, the blood vessels usually regress; however, in tendinopathy, excessive blood vessels resulting from prolonged VEGF overexpression impair the tendon’s biomechanical properties, promote scar tissue formation and inflammation, and delay the healing response [30,72,73]. In the context of tendon repair, the overexpression of VEGF can result in poor and abnormal vasculature, commonly referred to as “hyperpermeable”. These vessels are leaky and incapable of providing adequate oxygen and nutrients, thereby hindering proper tissue regeneration [74]. The prolonged presence of blood vessels and high levels of VEGF can also lead to the upregulation of MMPs and scar formation, further impairing tendon repair (Table 1) [33].

To facilitate tendon repair, it is crucial to regulate angiogenesis and VEGF at an early stage (Table 1) [30,32]. The presence of blood vessels and high levels of VEGF for extended periods can have detrimental effects [30,72]. Therefore, an alternative strategy is to stabilize the neovessels, which can provide adequate oxygen and nutrients to support tendon regeneration [74].

Tendon injury leads to the production of HIF-1α, resulting in the overexpression of VEGF [71,73]. While this process can aid in tendon repair, in the case of chronic tendon disorders, prolonged angiogenesis can weaken the mechanical strength of the tendon by breaking down the surrounding tissue through VEGF-triggered MMPs and upregulation of the hypoxia-associated apoptotic gene BNIP3. This leads to poor matrix remodeling and cell death [17,30,31]. Studies have demonstrated that the regulation of hypoxia can promote tendon repair. Tendon repair can be enhanced by regulating the HIF-1α/VEGF/BNIP3-mediated pathway (Figure 3).

## 3. Oxidative Stress

Nicotinamide adenine dinucleotide phosphate (NADPH) oxidase (NOX) is a multi-subunit protein complex responsible for generating ROS, including hydrogen peroxide (H_2_O_2_) and hydroxyl radicals [75]. These enzymes play crucial roles in several biological processes, such as host immunity, cell signaling, and maintaining tissue homeostasis [76]. All NOX isoforms transfer two electrons from NADPH to molecular oxygen, generating superoxides (O_2_^•−^) and H_2_O_2_ [77]. Various factors trigger the production of ROS by NADPH, including mechanical factors, hypoxia, cytokines, angiogenic factors, and transforming growth factor beta [78,79,80]. An imbalance between ROS and antioxidants can lead to the excess formation of ROS, which can cause disturbances in biological processes by targeting and damaging biomolecules within a cell, such as nucleic acids, proteins, and lipids. Excess ROS can render proteins susceptible to proteolytic degradation by altering their electric charge, by cross-linking proteins, and due to protein carbonylation. Conformational changes within proteins can result in the inhibition of enzymatic activities, increased susceptibility to proteolysis, and altered immunogenicity [75,81]. These modifications can contribute to a range of clinical pathologies, including mitochondrial dysfunction, musculoskeletal damage, and impaired muscle regeneration [82,83,84,85]. Excessive ROS levels can lead to damage to the musculoskeletal system, resulting in decreased myoblast function, increased myoblast apoptosis, and impaired muscle regeneration [83,84,85]. ROS have been implicated in several musculoskeletal disorders, including rheumatoid arthritis, osteoporosis, and tendinopathy, and may disrupt the integrity of mesenchymal stem cells by interfering with cellular proliferation, differentiation, and apoptosis (Table 2) [86,87]. Although limited research has focused specifically on oxidative stress and tendon disorders, it is believed that mechanical overload, overuse, and other comorbid factors can stimulate ROS production, leading to tendon damage [88,89,90]. Antioxidant therapy has shown promise in alleviating tendinopathy in in vitro and in vivo studies, suggesting that regulating oxidative stress may be a viable treatment option for tendon disorders (Table 2) [20,21,22,23,24].

### 3.1. Oxidative Stress Inhibition Promotes Cell Viability In Vitro

In vitro studies have demonstrated that antioxidant treatment can protect against excessive ROS levels (Table 2) [20]. To simulate the physical environment of oxidative stress, researchers have developed a chemically induced (H_2_O_2_) in vitro model [75,76]. The antioxidant capacity of dehydroepiandrosterone (DHEA) in promoting tenocyte viability has been documented. DHEA, a circulating steroid abundantly present in the body, has previously been known to function as an antioxidant and promote musculoskeletal health [91]. Mukohara et al. conducted a study in which DHEA treatment promoted tenocyte viability with minimal cytotoxicity and suppressed the overproduction of ROS [92]. Peroxiredoxin-5 (PRDX5) is a well-known antioxidant with six identified isoforms, all of which play important roles in neutralizing and eliminating H_2_O_2_ and other oxidizing molecules. Previously, PRDX5 was shown to be highly elevated in human tendinopathic samples when compared to normal healthy tendons, indicating that a high expression of PRDX5 counterbalances or neutralizes high levels of H_2_O_2_ or O_2_^•−^ induced oxidative stress [93]. In human tenocytes subjected to oxidative stress conditions (H_2_O_2_ exposure), the PRDX5 levels were also evaluated. The overexpression of H_2_O_2_ promotes PRDX5 synthesis, which protects against oxidative stress by significantly reducing H_2_O_2_-induced tenocyte apoptosis and improving collagen synthesis [94].

Diabetes mellitus increases the risk of musculoskeletal disorders, such as diabetic amyotrophy, tendinitis, and tendon ruptures. It can also impair normal tendon healing and make patients prone to tendinopathy [95]. A high-glucose environment has been reported to promote the production of ROS; therefore, oxidative stress may play a role in diabetes-mellitus-associated pathologies. Kurosawa et al. evaluated the effects of apocynin (an inhibitor of NOX) in vitro using a tenocyte model of high-glucose-induced oxidative stress. Apocynin treatment reversed the effects of high-glucose conditions by significantly reducing the levels of NOX1, NOX4, and IL-6, and increasing tenocyte viability. These findings suggest that apocynin may be a viable prodrug for treating diabetic tendinopathy [96].

Advanced glycation end products (AGEs) are toxic compounds that are formed under hyperglycemic conditions and can lead to tissue damage and organ dysfunction [97]. The accumulation of AGEs can impair the healing process and cause serious damage to organs and tissues [98]. AGEs can also interact with RAGEs and activate NADPH, leading to the generation of excess ROS [99]. AGE-associated oxidative stress has been shown to increase intracellular levels of H_2_O_2_ and O_2_^•−^ ions. Previous studies have also implicated the role of AGEs in tendon disorders, as AGEs accumulate in proteins such as collagen in the bones and tendons, leading to altered biomechanical properties and the disruption of tendon homeostasis. AGEs may also induce negative effects by promoting oxidative stress [100,101]. Furukawa and colleagues reported the antioxidant effects of apocynin on AGE-induced oxidative stress in human rotator-cuff-derived cells. Apocynin reduced the expression of NOX1, IL-6, and AGEs and suppressed NOX activity, leading to a significant reduction in ROS and improved cell viability of the rotator-cuff-derived cells [102]. These studies suggest that apocynin may be a potent treatment for alleviating oxidative stress and improving tendon health.

DHEA is a natural circulating hormone that is a precursor of sex steroid hormones, which are converted into testosterone and estradiol [103]. In the musculoskeletal system, DHEA promotes muscle strength and growth while providing protection against oxidative stress by inhibiting NOX. Additionally, low levels of DHEA have been associated with an increased risk of developing diabetes mellitus, as patients with type 2 diabetes have low serum DHEA levels. In vivo studies have also shown that the administration of DHEA reversed the effects of hyperglycemia in a type 2 diabetes mouse model [104]. The effect of DHEA treatment was evaluated in an in vitro and in vivo study that recapitulated hyperglycemia-induced tendon pathology. DHEA provided protection against HG-induced oxidative stress in tenocytes by inhibiting NOX1 and preventing apoptosis. Furthermore, in a diabetic tendinopathy model, DHEA decreased the expression of inflammatory cytokines and MMPs but increased collagen 1 expression, suggesting that it may play a role in promoting the healing process [92].

Autophagy is a critical mechanism for maintaining cellular homeostasis, allowing the recycling and degradation of dysfunctional cellular components. ROS are activated by various intrinsic and extrinsic factors, including ROS accumulation. Autophagy has been documented to maintain the oxidant–antioxidant balance by eliminating excess ROS and has been shown to prevent ROS accumulation [105]. In a study by Chen and colleagues, H_2_O_2_ treatment led to increased ROS expression and the reduced proliferation and colony-forming capacity of human TDSCs [105]. However, treatment with rapamycin and starvation, known activators of autophagy, reversed the effects of H_2_O_2_ treatment through its antioxidant capacity, as observed by the improved differentiation and high stemness marker expression of the TDSCs. These findings suggest that autophagy plays a crucial role in the prevention of oxidative-stress-induced damage [106].

Hyaluronic acid, which has been suggested as a treatment for tendon disorders and has strong antioxidant potential, protects against oxidative-stress-induced apoptosis in tenocytes by reducing the activation of Caspase 3 and Caspase 7 (stimulators of apoptosis). Hyaluronic acid treatment also significantly improves cell viability [107]. Similarly, in a study by Gallorini et al., hyaluronic acid administration provided protection against oxidative-stress-related cytotoxicity and reduced Nrf2 expression [108]. The same group reported the combined effects of hyaluronic acid with Methylsulfonylmethane (MSM), which promoted protection against H_2_O_2_-induced cytotoxicity and ECM remodeling [109].

NMN is another important antioxidant that has been studied for its potential to protect against tendinopathy. In vitro and in vivo studies by Yamaura and colleagues showed that NMN has antioxidant effects against tendinopathy and promotes the expression of SIRT 1 and SIRT 6, known for their anti-aging and anti-inflammatory roles, while reducing the expression of NOX1, NOX4, Il-6, and ROS [22].

Taurine has also been studied for its antioxidant potential to protect against mitochondrial-dysfunction-generated ROS. Ueda et al. found that taurine administration improved cell viability by reducing oxidative stress and cellular apoptosis in human tenocytes exposed to H_2_O_2_ [110].

While these studies highlight the potential of antioxidants in promoting tenocyte health and function under induced oxidative stress (Table 2), in vitro studies cannot fully recapitulate the mode of action through which these antioxidants induce their positive effects. In vivo studies may provide a more comprehensive insight into antioxidant activity and offer a better understanding of the pathophysiology of oxidative-stress-induced tendon disrepair.

### 3.2. Antioxidants’ Role in Maintaining Cellular Integrity In Vivo

Animal models of oxidative stress may replicate the deleterious effects of oxidative stress on the musculoskeletal system. Oxidative stress leads to tissue damage due to its accumulation in the organs, but excess accumulation is prevented by the activity of Superoxide dismutase (SOD), a crucial antioxidant that maintains the oxidation–reduction balance. The absence of SOD has been shown to have adverse effects on the musculoskeletal system [111]. To gain a deeper understanding of the role of SOD in maintaining enthesis integrity, Morikawa et al. developed an SOD-deficient mouse model. The model highlighted how the absence of SOD can result in severe histopathological changes at enthesis, which resemble some features observed in rotator cuff degeneration, such as increased intracellular ROS and decreased collagen 1 expression. SOD1^−/−^ mice exhibited abnormalities in the fibrocartilaginous zone, displayed impaired tissue elasticity, and had decreased mechanical properties. The model accurately recapitulated the tendon/enthesis degeneration observed in humans [23].

In addition to SOD and other naturally synthesized antioxidants, various dietary antioxidants have been reported to reduce oxidative stress and promote repair. Vitamin C, a well-documented ROS scavenger, is naturally present in some foods because humans are unable to synthesize it endogenously. Vitamin C can also work as a co-factor for collagen synthesis, suggesting that it can play a crucial role in promoting tendon healing [112]. Morikawa and colleagues evaluated the effects of Vitamin C administration on the alleviation of histopathological changes in SOD1^−/−^ mice. Vitamin C supplementation improves the histological parameters of enthesis by promoting chondrocyte number and chondrocyte and collagen fiber alignment and maintaining the integrity of the fibrocartilaginous zone [21].

Uehara et al. evaluated the levels of SOD in a rat model of rotator cuff tear and observed a significant decrease in SOD expression compared to the rotator cuff injury model, highlighting the importance of SOD in preventing tendon degeneration [113].

Szeto-Schiller-31 (SS-31) or Elamipretide a known mitochondrial protectant that has been shown to restore mitochondrial function by enriching phospholipid cardiolipin within the mitochondrial inner membrane. [114]. Cardiolipin makes up 10–20% of the total lipids within the mitochondrial inner membrane and is an important component for mitochondrial function. Alterations in cardiolipin biogenesis and distribution can lead to mitochondrial dysfunction and excess ROS production, which can in turn oxidize cardiolipin, resulting in mitochondrial dysfunction. Treatment with SS-31 has been shown to reverse these effects [115]. Although studies have implicated cardiolipin oxidation and mitochondrial dysfunction in a number of diseases, there are limited studies detailing their roles in the musculoskeletal system. Zhang and colleagues used SS-31 to promote mitochondrial function and enhance tendon healing in a murine tendinopathy model. The tendinopathy model showed a significant reduction in SOD expression, mitochondrial dysfunction, and impaired tendon histopathological features. However, the administration of SS-31 significantly improved mitochondrial function, tendon structure and biomechanical function, and SOD levels, suggesting that it plays a role in tendon healing [116].

SOD is an enzyme well known for its role in maintaining oxidative balance. It is located in the mitochondrial matrix and catalyzes the transformation of superoxide into oxygen and hydrogen peroxide. Therefore, SOD inhibition results in a decreased capacity to scavenge superoxide radical anions, which can lead to free radical damage to mitochondrial components and subsequently result in mitochondrial dysfunction [117]. Using a murine supraspinatus tendinopathy model, researchers found a significant decrease in SOD gene expression and activity. However, following the removal of subacromial impingement, the levels and activity of SOD significantly increased, suggesting that mitochondrial dysfunction plays a role in promoting tendinopathy [118]. Similarly, in another study, the SOD levels were significantly downregulated in a rat rotator cuff injury model. SOD downregulation may contribute to oxidative stress, leading to poor tendon healing [119]. Treatment with antioxidants can promote SOD expression and reduce oxidative stress in animal models. Treatment with quercetin was shown to enhance the expression of glutathione peroxidase (GPx) and SOD and to reduce the extent of tendon adhesion, making it a viable treatment for preventing tendon adhesion [120]. Furthermore, hydrogen water, which has been suggested to exhibit antioxidant properties, was investigated for its ability to reduce tendon adhesion. Treatment with hydrogen water helped reduce tendon adhesion and promoted the expression of antioxidants, including SOD, glutathione (GSH), and Nrf2, compared to the saline group [121].

**Table 2:** Antioxidant treatment promotes tendon repair and counters the negative effects of ROS imbalance. ↑: upregulation; ↓: downregulation.

Study Models	Study Purpose	Study Outcome	Observed Changes	Ref.
In-Vitro studies	DHEA role in regulating ROS levels in vitro	DHEA treatment suppressed ROS production without any cytotoxicity	DHEA treatment: ROS ↓	[92]
	Determine protective effects of PRDX5 against OS-induced apoptosis in tenocytes	PRDX5 promoted collagen synthesis and provided protection against OS-induced apoptosis		[94]
	Determine the effects NOX inhibitor apocynin on cell viability	Treatment using apocynin promoted cell viability due to the downregulation of NOX1, NOX4, and IL-6	Apocynin treatment: IL-6 ↓ NOX1 ↓ NOX4 ↓	[96]
	Protective effect of apocynin against OS-exposed rotator cuff tendon cells	Apocynin may promote tendon health due to the downregulation of NOX and IL-6	Apocynin treatment: IL-6 ↓ NOX1 ↓	[102]
	Effects of H_2_O_2_ exposure on TDSCs and the involvement of autophagy in regulating oxidative stress	H_2_O_2_ exposure caused TDSCs to lose their self-renewal and stemness markers. Treatment using rapamycin reversed these effects	H_2_O_2_ Exposure: OCT4 ↓ Nanog ↓ NS ↓ SSEA-4 ↓ Rapamycin treatment: OCT4 ↑ Nanog ↑ NS ↑ SSEA-4 ↑	[106]
	Antioxidant potential of hyaluronic acid in vitro	Hyaluronic acid promotes cell viability by protecting against OS induced cellular apoptosis	Hyaluronic acid treatment: Caspase 3 ↓ Caspase 7 ↓	[107]
	Protective effects of hyaluronic acid against OS-induced cytotoxicity	Hyaluronic acid provides protection against OS-induced oxidative stress in rotator cuff tendon cells	Hyaluronic acid treatment: Nrf2 ↓	[108]
	Effect of nicotinamide mononucleotide treatment against in vitro tendinopathy model	Through its antioxidant capacity, NMN provides protection against tendinopathy due to the downregulation of NOX, ROS, and IL-6	NMN treatment: IL-6 ↓ NOX1 ↓ NOX4 ↓ SIRT ↑	[22]
In-vivo studies	Determining the effects of lack of SOD in a SOD-deficient mouse	Absence of SOD caused a significant decrease in biomechanical strength and abnormal fibrocartilaginous zones	SOD^−/−^ mice: Enthesis impairment	[23]
	Positive effects of Vitamin C in a SOD^−/−^ mice model	Vitamin C treatment promoted chondrocytes numbers, the alignment of chondrocytes, and proper alignment of collagen fibres	Vitamin C treatment: Improved enthesis integrity	[21]
	Determine levels of SOD in a SOD-deficient mice model	SOD was significantly downregulated in rotator cuff tear mice model	SOD^−/−^ mice: SOD ↓	[112]
	Evaluate the effects of mitochondrial protectant SS-31 in a mouse supraspinatus tendinopathy model	SS-31 treatment improved the biomechanical function of tendons and upregulated SOD levels	SS-31 treatment: SOD ↑ ROS ↓	[115]
	Evaluate therapeutic effects of quercetin a rat Tendinopathy model	Quercetin treatment promoted the expression of antioxidants and decreased the levels of inflammatory cytokines and MMPs	Quercetin treatment: MMP ↓ ICAM-1 ↓ HO-1 ↑ Nrf2 ↑	[117]
	Evaluate the anti-oxidative effects of DHEA in an oxidative stress animal model	DHEA treatment improved tendon matrix turnover via its anti-inflammatory effects	DHEA treatment: MMP2 ↓ TIMP2 ↓ Collagen 3 ↓	[92]
	Effect of NMN treatment in rat tendinopathy model	NMN treatment promoted tendon repair by downregulating oxidative-stress-associated NOX1 and NOX4	NMN treatment: NOX1 ↓ NOX4 ↓ SIRT1 ↑ SOD ↑	[22]
	Anti-oxidative effects of epigallocatechin gallate loaded on hyaluronic acid hydrogels in a rat tendinopathy model	The treatment mitigated the tendinopathy changes by improved histology scoring and promoting collagen expression	Treatment response: Collagen 1 ↑ Collagen 3 ↓ PPARr ↓ SOX9 ↓	[118]
	Evaluate the ROS scavenging capacity of CeO_2_ nanoparticles in a rat tendinopathy model	CeO_2_ NPs promoted tendon repair by alleviating ROS and activating Nrf2	CeO_2_ treatment: ROS ↓ Nrf2 ↑	[119]
Clinical studies	Levels of PRDX5 in tendinopathic samples	PRDX5 was abnormally expressed in degenerated human tendon samples	Tendinopathic tendon: PRDX5 ↑	[93]
	Compare the levels of oxidative stress markers in pre- and post-operative tendinopathy patients	Oxidative stress markers were significantly elevated in pre-operative patients as opposed to post-operative	Pre-operative patients: Nrf2 ↑ Total oxidant status↑	[121]
	Determine oxidative-stress-related structural abnormalities in elite soccer patients	Elevated oxidative stress markers were found in elite soccer player patients. Oxidative-stress-associated structural abnormalities were observed in patients	-	[89]

DHEA, Dehydroepiandrosterone; PRDX5, Peroxiredoxin-5; ROS, Reactive oxygen species; NOX, NADPH oxidase; NOX1, NADPH oxidase 1; NOX4, NADPH oxidase 4; IL-6, Interleukin-6; Hydrogen peroxide, H_2_O_2_; TDSCs, Tendon-derived stem cells; OCT-4, Octamer-binding transcription factor 4; NANOG, Homeobox protein NANOG; NS, Nucleostemin; SSEA-4, Stage-specific embryonic antigens-4; OS, Oxidative stress; Nrf2, Nuclear factor erythroid 2–related factor 2; NMN, Nicotinamide mononucleotide; SOD, Superoxide dismutase; SS-31, Elamipretide; ICAM-1, Intercellular adhesion molecule; HO-1, Heme Oxygenase 1; MMP2, Matrix metalloproteinase 2; TIMP2, Tissue inhibitor of matrix metalloproteinase 2; SIRT1, Sirtuin 1; SIRT, Sirtuin; PPARr, Peroxisome proliferator-activated receptor; SOX9, SRY-Box Transcription Factor 9; CeO_2,_ Cerium Oxide.

Flavonoids, such as quercetin, are examples of dietary antioxidants that have been shown to possess multifunctional effects on a variety of cells, including cardiomyocytes; neurons; and kidney, tendon, and liver cells. Quercetin has been found to downregulate NOX, MMPs, and Collagen-3, and upregulate collagen 1 in hyperglycemia-induced oxidative stress tenocytes [122]. Additionally, Semis and co-workers assessed the therapeutic effects of quercetin in a collagenase-induced tendinopathy model, where quercetin treatment prevented ECM degradation by suppressing the levels of MMP, decreased the levels of the inflammatory marker ICAM-1, and promoted the expression of the antioxidants HO-1 and Nrf2 [123].

Mukohara and co-workers also evaluated the protective effects of DHEA in a diabetic rat model, where DHEA treatment downregulated the inflammatory marker IL-6 and suppressed the expression of MMP2 and collagen 3. Furthermore, it promotes tendon matrix synthesis by upregulating the collagen 1 gene. Although the exact mechanism by which DHEA induces its antioxidative effects is unknown, researchers have proposed that it may help alleviate certain pathophysiological symptoms of diabetes-induced tendinopathy in humans [92].

Yamaura et al. further expanded this idea by administering NMN to promote tendon repair in a collagenase-induced tendinopathy model [22]. NMN treatment provided protection against OS-induced damage by upregulating SIRT1 and SOD activation while downregulating NOX1 and NOX4. Future animal studies should focus on the mode of action by which NMN stimulates tendon healing in animal studies [22].

The utilization of HA hydrogels loaded with antioxidant agents has been demonstrated to significantly mitigate oxidative stress and promote tendon healing. For example, Hsiao and colleagues developed a HA hydrogel impregnated with an antioxidant, epigallocatechin gallate, to evaluate its impact on a collagenase-induced tendinopathy model. This HA hydrogel facilitates the healing process and diminishes tendinopathy changes by decreasing oxidative stress [124]. Another study demonstrated that epigallocatechin gallate mitigated the deleterious consequences of H_2_O_2_-induced inflammatory responses in tenocytes. Epigallocatechin gallate treatment notably attenuated the H_2_O_2_-induced cytotoxicity by diminishing the expression of inflammatory-associated proteins, including inducible nitric oxide synthase (iNOS), nuclear factor kappa B (NF-κB), and cyclooxygenase-2 (COX-2), while augmenting the expression of type I collagen [125].

Researchers have also developed artificial enzymes for ROS scavenging. One example is the use of cerium oxide nanoparticles (CeO_2_ NPs), which possess potent ROS scavenging capacities. In a study by Xu et al., CeO_2_ NPs provided antioxidant support by lowering the levels of ROS. CeO_2_ NPs offered protective effects in a collagenase-induced tendinopathy model by activating Nrf2 [126]. Nrf2 is believed to play a crucial role in providing protection against oxidative stress by balancing the oxidation and anti-oxidation levels. In addition, Nrf2 is thought to be important in tendon growth and regeneration. Consequently, the activation of Nrf2 by CeO_2_ NPs may stimulate antioxidation and promote tendon healing [127].

These in vivo studies underscore the importance of antioxidants in maintaining oxidative homeostasis within the musculoskeletal system (Table 2). Through unidentified pathways, antioxidants can provide relief against OS-induced tissue damage and enhance overall tendon health.

### 3.3. ROS and Antioxidant Imbalance Promotes Tendinopathy

Based on the available evidence, oxidative stress is considered an important factor in mediating chronic tendon disorders (Table 2). While the exact mechanisms leading to oxidative stress-induced tendon damage are not yet fully understood, it is believed that a cascade of different events, including mitochondrial dysfunction and the upregulation of NOX, results in the excess accumulation of ROS. Despite this, limited clinical studies have been conducted to investigate the role of oxidative-stress-related genes in tendinopathy, and further research is needed to determine the genes responsible for negatively affecting tendon health.

Studies have indicated that high levels of oxidative stress may play a role in tendinopathy by disrupting the levels of the antioxidant PRDX5 [93]. Furthermore, research has shown that serum oxidative stress markers are significantly upregulated in patients with degenerated rotator cuff tears [128].

Hence, impaired oxidative stress plays a role in the development of tendinopathy. A recent study compared superoxide-induced oxidative stress levels between individuals with and without rotator cuff tears. Based on dihydroethidium (DHE) relative fluorescence intensity, investigators found that the superoxide-induced oxidative stress was notably higher in patients with rotator cuff tears. This observation indicates that oxidative stress may contribute to tendon degeneration [129]. In addition, a study by Abate et al. found that elite soccer players with tendon structural abnormalities also exhibited high levels of oxidative stress markers. Their findings suggest that high oxidative stress levels may lead to a deficit in SOD levels and promote tendon damage [89].

According to the current body of knowledge, reactive oxygen species (ROS) are consistently generated in injured tendons. The resulting accumulation of ROS causes a disparity between ROS and antioxidants, leading to oxidative stress [15].

### 3.4. Oxidative-Stress-Associated Genes Promote Tendinopathy

One of the defining characteristics of tendinopathy is persistent, non-resolving chronic inflammation, which is characterized by increased levels of pro-inflammatory markers. This inflammation is typically triggered by the activation of the interferon and NF-κB pathways [130]. Among these pathways, NF-κB has gained particular attention for its potential therapeutic applications in the treatment of chronic tendon disorders. Previous research has suggested that oxidative stress can activate NF-κB [131]; it has also been shown to exhibit both pro-oxidant and antioxidant activity. However, in tendinopathy, NF-κB is upregulated, leading to a continuous inflammatory response that impedes the tendon repair process.

Recent studies have suggested that targeting the IKKβ/NF-κB pathway is a promising therapeutic strategy for the treatment of chronic tendon disorders. Researchers have found that the genetic deletion of IKKβ can partially protect mice from chronic overuse-induced tendinopathy, and an IKKβ knockout model has shown significant improvements following surgical repair [132]. Another study explored the role of NF-κB in TDSC senescence, a feature of tendinopathy, and found that inhibiting NF-κB could alleviate TDSC senescence and aging-associated inflammation [133]. Therefore, inhibiting oxidative stress might help prevent the overexpression of NF-κB and promote an appropriate inflammatory response.

Previous research has also shown that ROS-induced NF-κB activation promotes the expression of COX-2, a protein that plays a crucial role in the production of prostaglandins, which are involved in mediating inflammation [134]. COX-2 is essential for regulating the inflammatory response, and recent studies have suggested that COX-2 inhibition can impair tendon bone healing. In a recent study, researchers observed that using a COX-2 inhibitor impeded tendon bone healing following anterior cruciate ligament reconstruction due to the significant downregulation of prostaglandin E2 [135].

A study demonstrated that the use of the non-steroidal anti-inflammatory drug (NSAID) licofelone enhanced the process of tendon repair by inhibiting COX-1, COX-2, and 5-LOX. This inhibition reduces fatty infiltration and fibrosis [136]. However, a recent systematic review by Seah et al. reported that non-selective NSAIDs do not affect the rate of retear in clinical studies, whereas COX-2-selective NSAIDs can influence the rate of retear. Therefore, COX-2-specific NSAIDs should be used with caution following rotator cuff repair [137]. These findings suggest that the use of COX-2-specific inhibitors may impair the healing response and that further research is needed to understand how inhibition negatively affects tendon repair.

These findings suggest that oxidative stress contributes, in part, to tendinopathy. Future studies focused on identifying oxidative stress markers in human tendinopathy samples may provide a more comprehensive understanding of tendinopathy and aid in the development of more effective therapies to alleviate oxidative stress and tendinopathy.

## 4. Hypoxia and Oxidative Stress Play an Important Role in the Pathogenesis of Tendinopathy

### 4.1. Hypoxia Regulates Oxidative Stress in Chronic Tendon Disorders

**Figure 4:**
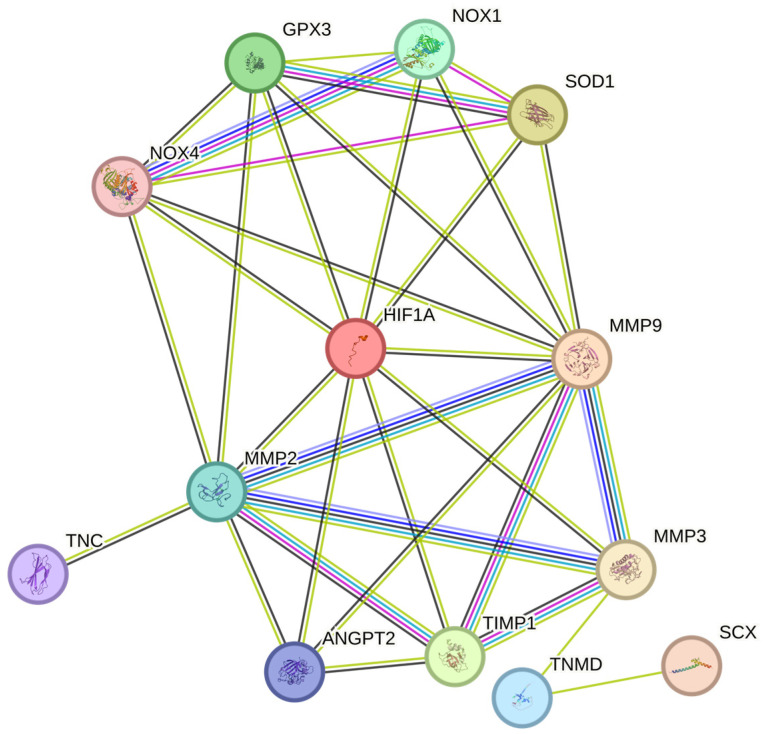
HIF-1α regulates ROS and worsens tendon health during TP. HIF-1α, Hypoxia-inducible factor 1-alpha; MMP-3, Matrix Metalloproteinase 3; SCX, Scleraxis; TNMD, Tenomodulin; TIMP1, Tissue inhibitor of metalloproteinases 1; ANGPT2, Angiopoietin 2; MMP-9, Matrix Metalloproteinase 9; MMP-2, Matrix Metalloproteinase 2; TNC, Tenascin C; NOX1, NADPH Oxidase 1; NOX4, NADPH Oxidase 4; SOD1, Superoxide dismutase 1; GPx3, Glutathione Peroxidase 3.

In conclusion, hypoxia and oxidative stress play a crucial role in the development and progression of tendinopathy (Table 1 and Table 2) [16,23]. The cumulative effects of these factors on tendon degeneration remain unclear. In hypoxic conditions, HIF-1 may modify the cytochrome chain, which is responsible for mitochondrial oxidative phosphorylation, resulting in reduced ATP synthesis and excess ROS formation [16,138]. This is supported by the fact that hypoxia leads to ROS production, which inhibits PHD activity, resulting in the stabilization of HIF-1α levels [139]. The accumulation of HIF-1α under hypoxia may be the result of the inhibition of antioxidants, which would lead to a loss of antioxidant capacity to scavenge excess ROS, thereby inducing systemic and local activation of HIF-1α. HIF-1α, in turn, activates target genes, such as erythropoietin and VEGF, which promote the production of ROS. Chronic hypoxia, via the accumulation of HIF-1α, inadvertently promotes NADPH oxidase activation, which intensifies ROS export and, in turn, promotes oxidative stress [140,141]. Following tendon injury, the upregulation of HIF-1α leads to excess ROS production, which results in incomplete tendon repair. Although the precise pathogenesis is not yet fully understood, it is speculated that the HIF-1α/ROS cascade may promote the progression of chronic tendon disorders by regulating several pathways [16,30,142]. We propose that HIF-1α regulates oxidative stress via the dysregulation of ROS and, in turn, the promotion of VEGF and other aforementioned factors. This cascade of mechanisms negatively affects tendon health and delays healing (Figure 4).

### 4.2. Chronic Hypoxia-Induced Oxidative Stress Delays Healing by Prolonging the Inflammatory Response

Chronic hypoxia-induced oxidative stress may impede tendon healing by disrupting the regulation of the NF-κB pathway [132,143]. NF-κB plays a crucial role in controlling cellular processes, including inflammation, cell survival, and stress response. Previous studies have indicated that NF-κB signaling is elevated in patients with rotator cuff tendinopathy. This increased NF-κB level plays a vital role in degenerative changes in the tendon [132]. Hypoxia-associated reactive oxygen species (ROS) cause an increase in NF-κB-associated inflammatory markers, such as TNFa, IL-1B, and PGE2. This inflammatory response is crucial for the removal of necrotic material and the production of collagen to promote tendon healing. However, a prolonged inflammatory response leads to tendon adhesion, which impairs the tendon repair process and negatively affects the tissue-resident cells [143]. Although the exact mechanism is still not fully understood, it is believed that the oxidative stress resulting from hypoxia activates NF-κB [144]. Under hypoxia, the rate of IKK degradation by IκB increases, leading to the activation of NF-κB and its translocation to the nucleus at a higher rate, where it upregulates inflammatory gene expression. Activated NF-κB further promotes HIF-1 levels, thereby enhancing HIF-1-mediated activity [145]. HIF-1, in turn, promotes the expression of NF-κB target genes, such as COX-2 [146] and IL-6 [147], resulting in a prolonged state of inflammation and delayed healing [105].

### 4.3. Hypoxia Promotes Oxidative Stress by Upregulating NOX and Downregulating Antioxidants

Following tendon injury, the expression of HIF-1 is promoted by hypoxia, which governs several metabolic processes, including oxygen homeostasis, energy metabolism, growth, and differentiation [148]. HIF-1 has been documented to promote the expression of the NOX family, particularly NOX1 and NOX4, which are highly elevated in oxidative-stress-induced tendinopathy animal models [92,96,149]. This suggests that a hypoxic environment promotes the expression of NOX members during tendinopathy.

Under typical physiological conditions, the reactive oxygen species (ROS) generated by NOX1 and other NOX isoforms regulate cell growth, differentiation, survival, apoptosis, metabolism, and migration by targeting the redox-sensitive cysteine residues in cellular molecules. Protein tyrosine phosphorylation (PTP) is a common molecule targeted by ROS within cells that controls the phosphorylation of numerous proteins involved in cellular signal transduction, potentially disrupting normal cellular homeostasis. Although the precise mechanism remains unclear, the mitogen-activated protein kinase system and phosphoinositide 3-kinase, which are regulated by the NOX family, including NOX1, have been shown to be activated by NOX4 in multiple studies [149,150].

The production of NOX4 has been documented to increase under hypoxia because of the autocrine activation of its mediator, transforming growth factor beta-1 [151]. Hypoxia promotes oxidative stress by stimulating NOX4 and ROS generation. NOX4 has also been shown to play a role in the chondro-osteogenic phenotype observed in tendon cells during tendinopathy. NOX4 activation under hypoxia promotes ROS expression, which then activates the ERK/JNK pathway, resulting in increased expression of SOX9 (chondrogenic marker) and RUNX2 (osteogenic marker). This indicates that NOX4-derived ROS under hypoxia play a role in the pathogenesis of tendinopathy [152] (Figure 4).

NOX1 expression has also been reported to be elevated during hypoxia. The upregulation of NOX1 is associated with an increase in HIF-1α and the stimulation of HIF-dependent target gene transcription. Consequently, an accumulation of H_2_O_2_ occurs in cells stably transfected by NOX1, which is believed to be a result of NADPH-dependent superoxide formation and HIF-1α activation [153].

Glutathione peroxidase 3 (GPx3) is a potent antioxidant that scavenges ROS and is a major contributor to the reduction of H_2_O_2_, hydrogen peroxides, and other oxidants. Elevated GPx3 expression has been documented to alleviate oxidative stress in tendinopathy, suggesting its potential as a therapeutic approach to treating this disorder [154]. Furthermore, GPx3 has a binding site for HIF-1, and its expression is regulated by HIF-1 following hypoxic exposure. However, owing to the accumulation of ROS resulting from NOX isoforms, GPx3 levels decrease over time, leading to a deficiency in antioxidants [155].

Superoxide dismutase 1 (SOD1) plays a crucial role in protecting cells from the detrimental effects of ROS, particularly superoxide anions. SOD1 specifically targets and neutralizes superoxide radicals by catalyzing the dismutation of two superoxide molecules into molecular oxygen (O_2_ and H_2_O_2_) [156]. The enzyme activity relies on a specific catalytic metal ion, which can be manganese (MnSOD), iron (FeSOD), nickel (NiSOD), or copper (Cu/ZnSOD). In eukaryotes, MnSOD (SOD2) and Cu/ZnSOD (SOD1) are documented isoforms [157]. Moderate overexpression of SOD reduces local hypoxia and prevents the accumulation of HIF-1. However, decreasing the levels of SOD via siRNA transfection resulted in an increase in the levels of superoxide ions and the accumulation of HIF-1α, which was reversed by using superoxide scavengers such as SOD [158]. In the case of chronic hypoxia, the ROS released by NOX may result in excess HIF-1α and ROS accumulation [159,160], resulting in the suppressed activity of SOD isoforms and the overall induction of a state of oxidative stress mediated by intermittent hypoxia.

Therefore, chronic hypoxia may promote oxidative stress (OS) and lead to poor tendon healing (Figure 4) [128,138]. Although the exact pathway is unknown, we speculate that the NOX family [92,96,128], which has been previously reported to regulate the levels of various MMPs [161], may delay tendon healing by increasing the levels of tendinopathy-associated MMPs such as MMP9 and MMP2 [161,162,163]. During tendinopathy, hypoxia promotes the expression of HIF-1α, NOX1, and NOX4. The overexpression of NOX isoforms results in the reduced expression of antioxidants such as SOD1 and GPx3, which inadvertently leads to excess ROS accumulation by the NOX family [152,153,154,164,165]. This, in turn, promotes the expression of various MMPs such as MMP2 and MMP9, and the downregulation of the MMP inhibitor TIMP1, resulting in excess ECM degradation [30,140,141,142]. Overall, the tendon repair process was impaired. (Figure 4)

Therefore, correction or partial modulation of the hypoxic response may be a feasible treatment approach to chronic tendon disorders. The regulation of HIF-1 would aid in maintaining the oxidant–antioxidant balance and promoting tendon repair by downregulating MMPs and ROS and ensuring appropriate ECM remodeling (Figure 4).

## 5. Summary

This review article examines novel therapeutic strategies for tendinopathy, with a focus on genes that may promote tendon healing. Specifically, we provide evidence regarding the role of impaired hypoxia-induced oxidative stress in chronic tendon disorders, as demonstrated by various pathways. However, the lack of clinical studies makes it difficult to identify a single target, and we argue that the combined influence of these factors is essential for the pathophysiology of tendinopathy.

The role of HIF-1 in tendons is crucial for understanding tendon health and tendinopathy development [16]. HIF-1 regulates oxygen balance and responds to hypoxia by controlling processes such as angiogenesis, erythropoiesis, and glycolysis (Figure 2) [41,42]. Owing to their limited vasculature, tendons are susceptible to chronic hypoxia, contributing to the development of tendinopathy [16,30]. In tendinopathy, HIF-1 is upregulated, which in turn activates its target genes and modulates the elevated expression of pro-inflammatory cytokines, apoptotic factors, and angiogenesis, exacerbating the condition [7,16,17,18].

Hypoxic preconditioning enhances the proliferative and differentiation capacities of tendon-derived stem cells (TDSCs) [29,49]. Transplanting hypoxic stem cells improves the histological and biomechanical outcomes in animal models [59]. Therefore, HIF-1’s role in the tendons encompasses regulation, implications for tendinopathy, and potential therapeutic avenues [16,31,51]

The dysregulation of HIF-1 leads to imbalances in the HIF-1/VEGF pathway, thereby promoting improper neo-angiogenesis [30,32]. While angiogenesis is beneficial for acute tendon injuries, excessive VEGF-driven angiogenesis can lead to issues such as hypertrophic scarring, elevated MMP levels, and decreased TIMP expression, negatively affecting tendon health [17,30,31]. Therefore, regulating HIF-1 may offer a viable strategy for tendon healing (Table 1, Figure 2).

Normally, HIF-1α is synthesized and degraded via ubiquitin-mediated proteolysis under normoxic conditions (Figure 1) [36], while hypoxia promotes HIF-1α expression and stabilizes its levels. However, chronic hypoxia inadvertently leads to negative effects [40].

As evidenced by previous studies, HIF-1 overexpression is linked to impaired pro-inflammatory responses, apoptosis, and excess angiogenesis, highlighting its potential as a therapeutic target (Table 1) [30,44]. HIF-1 overexpression promotes tendinopathy by inducing structural alterations, apoptotic gene expression, and inflammation [31,51]. Targeting HIF-1 or its downstream effects presents a potential approach to mitigating tendinopathy severity [16]. Further research is essential to uncover the precise mechanisms of hypoxia-mediated tendinopathy pathogenesis.

In addition to hypoxia, oxidative stress is another important physiological condition that has been implicated in musculoskeletal disorders (Table 2). Excessive ROS production can lead to oxidative damage to the biomolecules within cells, resulting in various clinical pathologies, including mitochondrial dysfunction, musculoskeletal damage, and impaired muscle regeneration [83,84,85]. ROS have also been implicated in musculoskeletal disorders, such as rheumatoid arthritis, osteoporosis, and tendinopathy [86,87], where they interfere with cellular functions, including proliferation, differentiation, and apoptosis [88,89,90].

In vitro studies have demonstrated the potential of antioxidant therapy to mitigate oxidative-stress-induced damage in musculoskeletal tissues (Table 2) [20]. Studies have shown that antioxidants such as DHEA [91,103], PRDX5 [93,94], NMN [22], hyaluronic acid [107,108], taurine [110], and quercetin [120,122,123] possess the ability to promote cell viability and protect against excessive ROS levels in tenocytes. In addition, in vivo studies have provided further evidence of the importance of antioxidants in maintaining oxidative homeostasis within the musculoskeletal system [23,110,111]. SOD, a crucial endogenous antioxidant [23], has been shown to play a protective role in enthesis, and dietary antioxidants such as Vitamin C have been found to alleviate oxidative stress and promote the healing of tendons [21].

Chronic hypoxia has been documented to lead to excess ROS production, causing oxidative stress. Indeed, previous studies have shown that NOX-derived ROS can be triggered by multiple factors including mechanical stress, hypoxia, cytokines, and growth factors [78,79,80]. Under hypoxic conditions, the balance between ROS production and antioxidant defense mechanisms is disrupted [16,23]. HIF-1 can modify the cytochrome chain, leading to reduced ATP synthesis and excess ROS formation [16,122]. The expression of NOX isoforms, particularly NOX1 and NOX4, has been observed to increase under hypoxic conditions, and these NOX isoforms contribute to the generation of ROS, which affects various cellular pathways [92,96,142,148,149,150,151]. Antioxidant defense mechanisms, such as SOD [138,139] and GPx3 [133,134], are compromised under chronic hypoxia, further exacerbating the oxidative stress within tendons. Therefore, for the treatment of tendinopathy, one therapeutic strategy might be to modulate hypoxia and, in turn, reduce oxidative stress, thereby improving tendon homeostasis (Figure 3 and Figure 4).

This study underscores the significance of maintaining oxidative homeostasis and suggests potential therapeutic strategies for alleviating chronic hypoxia and oxidative-stress-induced tendinopathy [Table 1 and Table 2]. Future research focused on understanding their mechanisms will help in devising effective clinical treatments for chronic tendon disorders.

Previous studies have established the paramount importance of oxidative stress and hypoxia in governing tendon health. However, the exact pathways through which these factors contribute to tendinopathy remain unclear. Further investigations into the common pathways for these factors will provide valuable insights into their pivotal role in the pathogenesis of tendinopathy.

## 6. Conclusions

Tendinopathy, a condition of multifaceted etiology, is characterized by impaired hypoxia and oxidative stress, both of which have detrimental effects on tendon health. The regulation of hypoxia following tendon injury may alleviate oxidative stress. This review examines the role of hypoxia-associated genes and oxidative-stress-related factors in tendon degeneration and provides evidence for novel therapeutic strategies to improve tendon health. This article is distinctive in its pathway-based evidence of the contribution of hypoxia-associated genes and oxidative-stress-related factors to tendon degeneration, and highlights recent advancements in promoting tendon healing in chronic tendon disorders. We believe that the evidence provided in this article can aid researchers in developing more effective treatment strategies for tendinopathy by targeting the aforementioned pathways/genes. The authors aimed to improve the understanding of tendinopathy and develop more effective treatment strategies for chronic tendon disorders by targeting these factors.
